# The Conservative Physiology of the Immune System. A Non-Metaphoric Approach to Immunological Activity

**DOI:** 10.1080/17402520600877216

**Published:** 2006

**Authors:** Nelson M. Vaz, Gustavo C. Ramos, Vitor Pordeus, Claudia R. Carvalho

**Affiliations:** 1 Departamento de Bioquímica e Imunologia ICB UFMG Belo Horizonte MG Brazil; 2 Departamento de Farmacologia ICB UFSC Florianópolis SC Brazil; 3 PROCEP – Hospital Pró Cardíaco Rio de Janeiro Disciplina de Reumatologia Fac Medicina, USP São Paulo SP Brazil; 4 Departamento de Morfologia ICB UFMG Belo Horizonte MG Brazil

## Abstract

Historically, immunology emerged as a biomedical science, concerned with host defense and production of anti-infectious vaccines. In the late 50s, selective theories were proposed and from then on, immunology has been based in a close association with the neo-Darwinian principles, such as random generation of variants (lymphocyte clones), selection by extrinsic factors (antigens)—and, more generally, on genetic determinism and functionalism. This association has had major consequences: (1) immunological jargon is full of “cognitive” metaphors, founded in the idea of “foreignness”; (2) the immune system is described with a random clonal origin, coupled to selection by random encounters; and (3) physiological events are virtually absent from immunological descriptions. In the present manuscript, we apply systemic notions to bring forth an explanation including systemic mechanisms able to generate immunological phenomena. We replace “randomness plus selection” and the notion of foreignness by a history of structural changes which are determined by the coherences of the system internal architecture at any given moment. The importance of this systemic way of seeing is that it explicitly attends to the organization that defines the immune system, within which it is possible to describe the conservative physiology of the immune system. Understanding immune physiology in a systemic way of seeing also suggests mechanisms underlying the origin of immunopathogeny and therefore suggests new insights to therapeutic approaches. However, if seriously acknowledged, this systemic/historic approach to immunology goes along with a global conceptual change which modifies virtually everything in the domain of biology, as suggested by Maturana.


*Clinical & Developmental Immunology*, June–December 2006; 13(2–4): 133–142

Taylor & Francis

Taylor & Francis Group

### The conservative physiology of the immune system. A non-metaphoric approach to immunological activity*

NELSON M. VAZ[1], GUSTAVO C. RAMOS[2], VITOR PORDEUS[3], & CLAUDIA R. CARVALHO[4]

[1]*Departamento de Bioquímica e Imunologia, ICB, UFMG, Belo Horizonte, MG, Brasil,* [2]*Departamento de Farmacologia, ICB, UFSC, Florianópolis, SC, Brasil,* [3]*PROCEP – Hospital Pró Cardíaco, Rio de Janeiro, Disciplina de Reumatologia, Fac Medicina, USP, São Paulo, SP, Brasil, and* [4]*Departamento de Morfologia, ICB, UFMG, Belo Horizonte, MG, Brasil*

**Abstract**

Historically, immunology emerged as a biomedical science, concerned with host defense and production of anti-infectious vaccines. In the late 50s, selective theories were proposed and from then on, immunology has been based in a close association with the neo-Darwinian principles, such as random generation of variants (lymphocyte clones), selection by extrinsic factors (antigens)—and, more generally, on genetic determinism and functionalism. This association has had major consequences: (1) immunological jargon is full of "cognitive" metaphors, founded in the idea of "foreignness"; (2) the immune system is described with a random clonal origin, coupled to selection by random encounters; and (3) physiological events are virtually absent from immunological descriptions. In the present manuscript, we apply systemic notions to bring forth an explanation including systemic mechanisms able to generate immunological phenomena. We replace "randomness plus selection" and the notion of foreignness by a history of structural changes which are determined by the coherences of the system internal architecture at any given moment. The importance of this systemic way of seeing is that it explicitly attends to the organization that defines the immune system, within which it is possible to describe the conservative physiology of the immune system. Understanding immune physiology in a systemic way of seeing also suggests mechanisms underlying the origin of immunopathogeny and therefore suggests new insights to therapeutic approaches. However, if seriously acknowledged, this systemic/historic approach to immunology goes along with a global conceptual change which modifies virtually everything in the domain of biology, as suggested by Maturana.

**Keywords:** *Immune system, organization, structural determinism, autopoiesis, Maturana*

### The history of immunology creates cognitive, defensive metaphors

The founding period of immunology (late XIX–early XXth centuries), under the influence of the germ theory and the development of vaccines (Pasteur 1878) is marked by other important medical inventions, such as the characterization of antibodies, human serotherapy with animal antitoxins, serological diagnosis of infectious diseases, and the first theory of antibody formation: Ehrlich's 1900 lateral chains theory. From this period on, anti-infectious protection granted by vaccines and serotherapy, became explained by unexamined "cognitive" metaphors, such as recognition, memory, etc.

The next period, marked mainly by immunochemical interests, centered in the study of antigens and antibodies and the appearance of template theories of antibody formation (Mazumdar 1996). It was also marked by a series of unexpected findings, such as the immunological nature of allergic reactions and anaphylaxis and the characterization of "natural" antibodies, such as human isohemagglutinins, apparently emerging spontaneously, without antigenic stimulation (Landsteiner 1901).

Correspondence: N. M. Vaz, Departamento de Bioquímica e Imunologia, ICB, UFMG, CP486, Belo Horizonte 30161-970, MG, Brasil. Tel: 55 31 3499 2640. E-mail: nvaz@ icb.ufmg.br

*Supported by CNPq-Brasil grant 30.5043/2003-0 to N. Vaz.

ISSN 1740-2522 print/ISSN 1740-2530 online © 2006 Taylor & Francis
DOI: 10.1080/17402520600877216

The late 1940s were marked by the first evidences of cellular participation in immunological phenomena (Chase 1945) and in the 1950s, immunology until then dominated by medical and biochemical interests, was suddenly invaded by biological issues. Lymphocytes were characterized as the substrate of "immunocompetence" (Gowans 1996). Tissue allografts were used and understood as immunogenic stimuli and the notion of "specific immunological tolerance" was forged in experiments of tissue transplantation (Billingham et al. 1953). Graft-versus-host (GvH) reactions were also characterized illustrating the powerful pathogenic potential of lymphocyte activation (Simonsen 1962).

The middle and late 1950s saw the emergence of the so-called "selective theories of antibody formation", inaugurating a strong and permanent union with neo-Darwinist Biology. According to the natural selection theory of antibody formation (Jerne 1955), "natural antibodies" arise spontaneously, without antigens, and are then "selected" and amplified by contact with specific antigens. A couple of years later Burnet (1957), suggests that lymphocyte "clones" arise spontaneously, without antigens, each one forming a single or a few antibodies and are then "selected" by antigens to undergo clonal expansion and antibody formation. The clonal selection theory provides a cellular basis for the induction of "allograft tolerance" in newborn mice, and Burnet suggest that a similar inhibitory phenomenon neutralizes (delete, inhibit) auto-reactive lymphocytes ("forbidden clones") preventing the immune system from harming the organism with "autoimmune diseases".

This set of propositions had the effect of locking theoretical immunology in a scenario that forbids the proposition of significantly different theories, because lymphocytes forbidden to interact physiologically with the organism and with other lymphocytes, cannot organize themselves in a system. Parallel important notions, such as the suggestion that antibody production followed cell selection and a multiplicity of not so strict specificities (Talmage 1959) were virtually ignored.

The net result of such a theoretic narrowness was the characterization of a massive variety of cellular/molecular components involved in immunological activity, together with a flagrant inability to create new vaccines, treat allergies or diagnose autoimmunity. In summary, immunological activity is described as resulting from the expansion/contraction/regulation of specific clones of lymphocytes; the recognition of "foreignness", i.e. the detection of the previously undetected materials, usually called self/non-self discrimination, is a guiding explanatory principle. This explanatory principle has a clear cognitive, metaphoric meaning (Tauber 1997) which we want to avoid and replace by an explanation based on the structural dynamics of the immune system.

### Explanatory principles and explanations

There is an important and generally unacknowledged difference between explanations and explanatory principles. Explanatory principles tend to hide that which they are supposed to explain, as if naming a problem would be equivalent to solve it. If we ask: "How are we aware of reality?" and someone answers that we are conscious human beings, consciousness becomes an explanatory principle that hides the problem of our awareness of reality. Criminals are frequently taken to be explanatory principles of crime, but actually crime has much more complex origins; to restrict crime, we must curtail the conditions that give rise to criminals. Gregory Bateson (1973) initiates one of his famous "metalogues" with his daughter ("What is an instinct?") saying that gravity is an explanatory principle that actually does not explain anything. Maturana (1987) also argues that explanatory principles are not explanations. Asking how the immune system recognizes "foreign" materials does not help us to understand how this recognition is done, but it has had the effect of making believe that this is what the immune system actually does. Yet, is it?

What makes an explanation? First, explanations are answers to special kinds of questions, questions that demand an explanation. Lectures can become boring when they answer questions, which were not made. Scientific explanations, the kind of explanations we are interested in science, always contain a generative mechanism, i.e. a collection of components and relations among components that, when operating, is able to generate for the observer the entity or the phenomenon he/she wants to explain. But generative mechanisms are not, in themselves, explanations because explanations are only configured when they are accepted by the listener, who may place many informal objections in his/her hearing. In short, explanations are answers that contain an acceptable generative mechanism able to generate whatever we want to explain. A final question is: when do we know that we have already explained what we want to explain? When the generative mechanism proposed is able to generate other (all the) phenomena in that particular domain of description (Maturana 1987).

Thus, what do we have to show to say that we have reached an explanation of immunological problems? We have to propose a mechanism able to generate all immunological phenomena, both physiological and pathological. Currently, the expansion/contraction/regulation of specific clones of lymphocytes is insufficient to generate all known immunological phenomena.

Immunological activity is based on an explanatory principle, called self/non-self discrimination or, as we prefer, the recognition of "foreignness", the detection of the previously undetected. This explanatory principle has a clear cognitive, metaphoric meaning

(Tauber 1997) which we want to avoid and replace by the structural dynamics of a lymphocyte network.

### Physiology and conservation

Immunological activity is currently described as an automatic molecular/cellular neo-Darwinian process (Silverstein 1999) based on a random origin of variants (lymphocyte clones) coupled to a process in which these clones are subsequently selected for action (activation/differentiation/expansion) by competition with other clones. Clonal selection is to immunology, what natural selection is to biology.

A random origin of variants is essential to maintain natural selection of living species as the guiding force in evolution because otherwise factors internal to the organisms would impose architectural restrictions to variation and become the important issues. Similarly, a random origin is essential to maintain clonal selection of lymphocytes as the explanation of immunological activity; otherwise, architectural restrictions, for example, the organization of lymphocyte networks (Jerne 1974; Vaz and Varela 1978) would become the important issues. Random processes are polarly opposite to conservative, systemic/historical processes. It is impossible to tell the story of random events and randomness fears to tread where a story is told.

Physiology is not a central concept in immunology, because it is historically linked with medicine and pathology. Different from genetics and biochemistry, which were born in the study of plants and animals, immunology was born as a branch of bacteriology, in the study of human infectious diseases. The idea of immune protection conferred to immunology a series of cognitive concepts, inherent in the notion of vaccines. Central notions such as immune recognition, memory and tolerance, are loaded with a cognitive meaning that remains unacknowledged and even unexamined (Vaz and Carvalho 1993; Tauber 1997). Although fundamental in current thinking, these notions are explanatory principles which we want to replace by structural concepts.

It is also easy to understand why conservation is not a central notion in neo-Darwinism, nor in current immunology, because conservation is unable to coexist with the random generation of variants believed to feed the subsequent selective process of lymphocytes, which is also believed to be driven by random encounters with immunogenic materials absorbed from the medium. However, as we shall now describe, there is solid evidence for conservation in immunological activity. We will discuss two particular issues of conservation: what became known as "oral (mucosal) tolerance" in adult animals (Faria and Weiner 2005) and the robust conservation of patterns of reactivity in natural immunoglobulins (Igs) (Nóbrega et al. 1993; Lacroix-Desmazes et al. 1999).

### Oral tolerance as conservation

Immunology lost a great opportunity to study physiological and conservative phenomena early in the XXth century, when different laboratories, both in Europe (Besredka 1909) and US (Wells 1911; Chase 1946), reported for the first time a phenomenon currently know as oral tolerance (Brandtzaeg 1996; Faria and Weiner 2005). Oral tolerance is usually interpreted as an inhibition of specific immune responsiveness to a protein immunogen, which is triggered by its previous ingestion as food. Actually, although this previous ingestion triggers a decrease in B and T cell responsiveness, it is not an inhibition but rather a stabilization or conservation of the level of specific responsiveness after a secondary parenteral immunization. This is made evident in animals which become "partially tolerant", i.e. which are significantly less responsive than controls, but still produce significant amounts of specific antibodies. These "partially tolerant" animals robustly maintain their level of specific responsiveness in spite of several successive immunizations with the specific immunogen (Verdolin et al. 2001).

This robust stabilization is the opposite of the progressive kind of responsiveness normally associated with the idea of immunological memory that is supposed to be on the basis of immune-protection by vaccination. And, although presently unappreciated, we claim that this is a fundamental aspect of immunological physiology.

The presence of dietary proteins is necessary to build a normal immune system. Mice maintained in conventional (non-sterile, non-SPF) environments and fed from weaning with a protein-free aminoacid-balanced diet, display several molecular, cellular and morphological abnormalities in their immune system (Menezes et al. 2003). The two major sources of immunogenic materials to which the organism is exposed are dietary proteins and products of the gut flora (the autochthonous microbiota). However, instead of immunizing the organism for progressive responsiveness, contacts with these materials lead to stable levels of specific responsiveness. Disturbances of the normal assimilation of food proteins and products of the gut flora lead to severe inflammatory gut diseases (IBD) (Duchman et al. 1996).

It is noteworthy that the parenteral injection of small doses (e.g. 10 $\mu$g) of proteins to which the animal is orally-tolerant triggers a strong inhibition of primary responses to unrelated antigens; the fact that secondary responses to these same immunogens are not similarly inhibited, indicates that this phenomenon cannot be explained by so-called "innocent bystander" mechanisms (Carvalho et al. 1996). This provides further support for the systemic (networkish) character of immunological activity.

### Natural, robust patterns of IgM production

An important recent development in immunology has been the development of methods allowing the assessment of Ig reactivity *en bloc*, by placing whole serum of normal (non-immunized) organisms in contact with complex mixtures of ligands, such as extracts of whole organs (muscle, liver, brain, etc.) or whole bacterial cultures, such as modified forms of immunoblotting (Nóbrega et al. 1993; Haury et al. 1994; Stahl et al. 2000) or "protein-chips" (Quintana et al. 2004). Results obtained with these methods have shown that patterns of reactivity of natural serum Igs are established early in ontogeny.

It may come as a surprise to learn that mice raised and maintained from birth in "antigen-free" conditions may robustly conserve standard concentrations (Hashimoto et al. 1978; Bos et al. 1986) and patterns of reactivity (Haury et al. 1997) in their naturally produced IgM, and also their rates of activation of T lymphocytes (Pereira et al. 1985). "Antigen-free" mice are immunologically abnormal: they form no germinal centers, do not develop lymph nodes, have no mucosal-associated lymphoid tissues and the synthesis of IgG and IgA (and probably IgE) is almost inexistent. However, they display normal numbers of IgM-forming cells in the spleen and their serum concentration of IgM is normal and display normal patterns of reactivity (Haury et al. 1997). This shows that fundamental aspects of immunological activity are internal to the organism and antigen-independent.

A large proportion of the Igs produced in the initial phase of ontogeny are multiconnected to other Igs and interference with their formation results in gross abnormalities in adult life (Marcos et al. 1986).

These results have shown that patterns of reactivity of natural serum Igs are established early in ontogeny and from then on are also robustly conserved throughout the healthy living of the organism (Mouthon et al. 1995; Lacroix-Desmazes et al. 1999). These patterns are influenced by genes important in the determination of immunological activity, such as the MHC complex and those coding lymphocyte clonal receptors (Vasconcellos et al. 1998).

In normal (non-immunized) organisms, the patterns of reactivity of IgG are also to a large degree conserved, when tested against extracts of autologous tissues, although not so much in relation to bacteria (Mouthon et al. 1995). An important observation is that these patterns of IgG reactivity may vary in predictable ways during severe diseases, such as autoimmune diseases and chronic parasitic diseases, both in humans (Ferreira et al. 1997; Stahl et al. 2000; Caligiuri et al. 2003; Fesel et al. 2005) and experimentally in animals (Fesel and Coutinho 1998; Vaz et al. 2000, 2001). These findings suggest that pathological processes are not random events and that the structural changes the organism undergoes during diseases follow courses that may be scrutinized by the analysis of serum Igs.

### Systems

The serious acceptance of notions such as the conservation of patterns of serum Igs has radical consequences. In all accepted versions of immunological theory, immunological activity stems from a collection of unconnected lymphocytes, but this is incompatible with the idea of conserved patterns of reactivity. Invariant relations among components, on the other hand, are essential in the organization of systems. A system is described as a collection of elements connected to each other in such a way that acting upon one element has repercussions upon all the others. This is not what current immunology accepts; the idea of lymphocyte networks (Jerne 1974; Vaz and Varela 1978) is no longer seriously discussed as a central concept. Except for brief moments of activating/inhibiting interactions, lymphocytes are believed to act independently from each other, i.e. to use the standard jargon, lymphocytes are supposed to respond specifically to stimuli.

According to Maturana a system is any collection of elements that through preferential interactions among themselves create an operational boundary that separate them from other elements, with which they can also interact and, thus, configure the medium in which this collection of elements (the system) operate as a totality. Therefore, a system exists as a totality in a medium with which it interacts and also exists in another domain: a structural domain, a space generated by the interactions among its components. In its totality, the system does not exist alone: it exists in a medium through interactions, which trigger structural changes in it. The system conserves its condition as a special kind of systems as long as the organization that defines it is conserved (Maturana 2002; Vaz et al. 2003).

In traditional immunology, the "immune system" is seen as a collection of lymphocytes which perform individually specific immune responses to immunogenic stimuli. But systems, on the other hand, are neither stimulated nor respond to anything. Dynamic systems may (actually, they must) undergo perturbations (changes of state) triggered by encounters with the medium in which they operate as such systems and by the flux of their own activity. These perturbations are compensated by changes in relations among components, otherwise the system loses its organization and is either destroyed or transformed into a system of another class. The term perturbation is not meant as irregular deviation from a normal path; actually, dynamic systems never exist in a non-perturbed condition. Living systems perform a ceaseless dance of perturbations and compensations in their structural drifting while conserving their characteristic self-maintained organization, which Maturana has named an autopoietic organization (Maturana 2002; Maturana and Poerksen 2004; Maturana 2005).

### Systems within systems

As a component of the organism of jawed vertebrates, the immune system is part of their physiology as living systems and participates in the maintenance of their autopoietic organization. As a sub-system component of the organism, the immune system has also an organization and a physiology of its own as a complex dynamic system. An important departure in this way of seeing is that the medium in which the immune system operates is the organism of which it is a component; the medium in which the organism operates remains as a meta-medium inaccessible to the immune system.

This is a contra-intuitive notion because immunologic activity is usually understood as immune responses to the contact with foreign antigens, and specific antibodies obviously react with these foreign materials. However, the fact that antibodies seem to be specifically directed to the antigens with which they react, is a fallacy which, to be understood, requires seeing the immune system in the two separate domains of description indicated by Maturana (2002). This double way of seeing is necessary to avoid the fallacy of instructive interactions.

### The fallacy of instructive interactions in immunology

A common misunderstanding of the nature of systems is that, as we see components of the system interacting with components of the medium and see that the system changes after these interactions, we may be mislead to believe that these interactions determine (guide, orient) the changes the system undergoes. This is known as the fallacy of instructive interactions (Maturana and Varela 1980, 1987). Systems are structure-determined entities, i.e. their changes are determined (guided, oriented) by their own structure. Actually, at each moment, it is the system's structure that determines which features (which components) of the medium may trigger perturbations in it. In immunology, the fallacy of instructive interactions arise as changes in the immune system which are described as specific immune responses, believed to be determined (guided, oriented) by interactions with (antigenic, immunogenic) components of the medium in which the organism operates.

### Specific antibodies as entities configured by immunological observations

Among several proposals concerning the understanding of living systems, perhaps the most radical aspect of Maturana ideas, is his definition of human "languaging", which he claims to be the basis of human understanding, from which derives his treatment of objective reality (Maturana and Mpodozis 1987; Maturana 1988). Language is usually understood as the transmission of symbolic information, but Maturana (1983) argues that the notion of information is unnecessary and bound to confuse the discussion of biological issues. He defines human languaging as a way of living (a ontogenic phenotype) typically human (Maturana and Mpodozis 2000), consisting of recursive coordinations of coordinations of consensual actions (Maturana 2002). The importance of actions in defining cognition is apparent in the title of one of his recent books: "From being to doing" (Maturana and Poerksen 2004). This definition of languaging as actions is also instrumental in his definition of reality; his aim is not to define what reality is, but rather to understand how we do what we do, including when we are asking what reality is. He claims that objects are configured through human actions (Maturana and Mpodozis 1987) in a kind of "inter-objectivity", which is not subjectivity. In other words, he never uses the notion of an objective independent reality as an explanatory principle.

We have recently exemplified how objects are configured in human actions by discussing the detection of "specific antibodies". Igs are described in the structural domain as components of the immune system and of the organism, which participate in its autopoietic organization. On the other hand, specific antibodies are functional entities distinguished in tests intentionally assembled to detect and quantitate them, and are supposed to define a domain of interactions between components of the immune system and components of the medium in which the organism lives. In this process, the intentionality of actions of immunologists operating in human languaging as observers of immunological activity, is transferred to the Igs detected as specific antibodies (Vaz and Ramos 2006). But the intentionality lies in the descriptions of immunologists; it is not present in the structural dynamics of the immune system.

According to Maturana (2006), "the immune system as a closed network of molecular and cellular productions that is part of the realization of the autopoiesis of an organism, does not protect or defend it. Defense and protection are metaphorical forms of describing the organism/niche relation that is being conserved in the lineage to which the observed organism belongs, that the observer proposes as a generative mechanism unaware of the process of phylogenic drift".

### An immune system?

As described next, current immunology is in theoretical turmoil. It is important to understand that our purpose is not finding answers to the problems and enigmas generated by the cognitive metaphors of traditional immunology, but rather to propose new notions which will necessarily generate other set of problems and enigmas.

If and when seriously considered, these notions raise a series of important questions. Which organization (invariant relations of components) is conserved and defines immune "systems" as special kinds of systems? In other words, if immunological activity arises in internal (closed) relations among lymphocytes, what is invariant (conserved) in these operations and how is this "internal" system "perturbed" by contact with its medium, which are not "external" materials (antigens), but rather the organism itself. Tracing an operational limit between the immune system and the organism is necessary for the definition of the immune system as a true system.

### Crisis in traditional immunology

Serological specificity, i.e. the capacity of a serum sample to react with a defined antigen X, named as anti-X, can be associated with Ig collections of widely different molecular composition. This "degeneracy" of Ig specificity has long been acknowledged as permissive (Talmage 1959), but may also be perceived as a major problem challenging basic tenets of immunological theory (Eisen 2001; Cohen and Sercarz 2004).

This conflict disappears when it is realized that specific antibodies and Igs are entities distinguished by different operations: antibodies are functional entities created (named) by the immunologist with practical (intentional, classificatory) interests, while Igs, viewed as components of the structural dynamics of the organism, are not oriented (directed, aimed) to react with (any) foreign materials, although they incidentally can be shown to do so, i.e. to behave as antibodies.

A second major issue pertains the concept of "natural tolerance" which deals with the interactions of the immune system with the organism. This problem has been confused for the last 50 years because the dominating theory—the clonal selection theory—forbids the reaction of lymphocytes with components of the organism ("forbidden clones"). Gradually, evidence in favor of a "physiological autoimmunity", different from the pathogenic autoimmunity prevailing in autoimmune diseases was acknowledged (Pereira et al. 1985; Coutinho et al. 1995; Coutinho 2005). This is an important point because since the simple presence of "auto-reactive" lymphocytes can no longer be incriminated as responsible for "autoimmune" aggressions and diseases, new triggering factors must be identified. What changes physiological into pathogenic autoimmunity?

As already pointed out, mucosal (oral) tolerance is the most frequent consequence of contact of the organism with the two major sources of external materials, namely: dietary proteins and products of the autochthonous microbiota. Therefore, in addition to "natural tolerance", which pertains interactions of lymphocytes with the organism, we must also consider mucosal tolerance as a major aspect of immunological activity.

It should be also acknowledged that, contrary to allograft tolerance in mice, which can only be induced in the neonatal period, the susceptibility to oral tolerance arises, grows and decays in parallel with immunocompetence (Vaz et al. 1997).

### Immanent immunopathogeny

A systemic view of immunologic activity may suggest how immunopathogeny arises. A frequent way of natural disassembling of systems is a loss of connections between system's components; machines frequently break down with this type of defect. In the immune system this would be equivalent to a loss of connectivity among lymphocytes and lymphocyte products, such as Igs and lymphocytes, for example, changes in the idiotype–anti-idiotype connectivity among B- and T-cells (Jerne 1974; Pereira et al. 1985; Marcos et al. 1986). This could lead to skewed profiles of expansion of a restricted variety of lymphocytes (oligoclonal expansions) which would mediate tissue damage.

It has been extensively demonstrated that T lymphocytes have the tendency to expand, sometimes to abnormally large proportions, when placed in lymphopenic organisms, in what was called homeostatic expansion or lymphopenia-induced lymphopoiesis (Troy and Shen 2003; Stockinger et al. 2004). These mechanisms probably play a natural role in the lymphocyte expansion of early periods of ontogeny, in which the first cells to emerge from primary organs find themselves in an organism free of lymphocytes (Min et al. 2003). Many of the Igs produced in this initial phase of ontogeny are multiconnected to other Igs and interference with their formation results in gross abnormalities in adult life (Marcos et al. 1986; Vakil et al. 1986).

If the initial population of T lymphocytes emerging in newborns is curtailed in its diversity, for example, by thymectomy performed at 3-days of age, the animal may develop a normal number of lymphocytes but develops autoimmune aggressions to various tissues and organs (Sakaguchi 2005). This happens because the resulting lymphocyte population remains oligoclonal (with a sub-optimal diversity) and expands to abnormal proportions. Thus, an immune system with a sub-optimal clonal (oligoclonal) composition may become pathogenic, regardless of developing a normal number of lymphocytes; clonal diversity is a neglected variable in the definition of immunological activity (King et al. 2004). Homeostatic proliferation of T cells following immunosuppression may actually represent a barrier to transplantation tolerance (Wu et al. 2004).

Actually, as discussed below, oligoclonal expansions of T cells are a common feature of numerous immunopathologic situations both in humans and experimental animals, including inherited immunodeficiency conditions, autoimmune and allergic diseases and also chronic parasitic infections. As suggested above, this form of malfunctioning may result from faulty connections between components of the system; isolated lymphocyte clones have a tendency to expand, and new forms of connectivity may drive the system into abnormal, skewed dynamics, which are manifested as immunopathology.

### IgE, eosinophils and T cell oligoclonality

Curiously and significantly, eosinophilia and increased IgE formation are frequently associated with the oligoclonal expansion of T cells in a large variety of situations, varying from inherited immunodeficiency conditions, autoimmune and allergic diseases and chronic parasitic infections.

Experimental allergic encephalomyelitis (EAE), is an immunologically-mediated condition of the central nervous system with destruction of myelin, which is considered an experimental model of autoimmunity similar to the human disease known as multiple sclerosis (MS). Its simplest form of induction requires the injection of adjuvants containing myelin or myelin basic peptide (MBP) into susceptible mouse strains; the disease is manifested by different degrees of motor dysfunction eventually leading to the death of the animal. Transgenic mouse strains containing exclusively MBP-specific T cells develop EAE "spontaneously" but this can be avoided by adoptive transfer of polyclonal T cells from normal compatible donors (Lafaille et al. 1994; Olivares-Villagomez et al. 2000). Thus, abolishing oligoclonality eliminate the pathogeny.

The same laboratory also reported experiments showing that IgE production is linked to T cell oligoclonality. Huge amounts of IgE (100-fold higher than normal) were produced by transgenic mice harboring one single T-cell clone and a single B-cell clone when they were injected with protein conjugates appropriate to stimulate (link) cells of the two clones. Thus, an extreme example of oligoclonality leads to extreme levels of IgE production. Similarly to the experiments with EAE, adoptive transfer of polyclonal T cells from normal compatible donors reduced the magnitude of IgE production (de Lafaille et al. 2001).

In parasitic diseases, an extensively investigated example of the association of an intense production IgE with T cell oligoclonality is the infection of Balb/c mice with *Leishmania major* (Launois et al. 1997; Pingel et al. 1999)

### Oligoclonality: Omenn's syndrome and a variety of other pathological situations

If the stability of profiles of reactivity of natural Igs reflects the operation of the immune system in healthy living, we may hope that specific alterations of these profiles are associated with particular pathologic conditions. As mentioned above, this has been actually noted in autoimmune and parasitic diseases both in humans and experimental animals. The mechanism of these alterations is unknown but they would be expected if some components of the immune system were isolated from the restrictions imposed by the network of interactions among lymphocytes and were independently expanded. In reality, there are an amazing number of pathologic situations associated with oligoclonal expansions of T lymphocytes. Omenn's syndrome is outstanding among these examples.

Omenn's syndrome is a severe, frequently fatal human immunodeficiency syndrome resulting from mutations in Rag1/Rag2 (Villa et al. 1999) in which pathogeny is linked to huge clonal expansions of CD4 + T lymphocytes and an intense production of IgE with eosinophilia; an inherited disorder characterized by an absence of circulating B cells and an infiltration of the skin and the intestine by activated oligoclonal T lymphocytes (Corneo et al. 2001).

Similar oligoclonal expansions are observed during GvH reactions following bone marrow transplants (Margolis et al. 2000; Orsini et al. 2000) and they eventually occur after blood transfusions (Wang et al. 1997) and congenital GvH (Appleton et al. 1994). Oligoclonal expansions of T cells are also present in several autoimmune diseases, such as lupus erythematosus (Murata et al. 2002), autoimmune thyroiditis (Sekine et al. 2000) and rheumatoid arthritis (Jendro et al. 1995; Guilherme and Kalil 2004) and also in diseases derived from distortions in maternal microchimerism, such as systemic sclerosis (Sakkas et al. 2002).

### IgE and persistent allergic sensitization

The association of lymphocyte oligoclonality with IgE production has been confirmed in the clinical scenario: VH gene usage in IgE responses of seasonal rhinitis patients allergic to grass pollen is oligoclonal and antigen driven (Davies and O'Hehir 2004). This association is also able to explain why the persistent production of specific IgE antibodies in mice requires intermittent repeated injections of small doses of antigen into strains genetically high-responders to this particular antigen; the interesting issue here is why higher doses of the same antigen will not succeed in inducing a persistent production of specific IgE in any mouse strain (Levine and Vaz 1970). We suggest that high-responder strains possess the few peculiar clones, which are able to detect and bind with sufficient affinity the minute doses of antigen to which the organism is intermittently exposed. These clones then expand oligoclonally. When higher doses of antigen are used, in any strain, the reaction involves many other clones and the oligoclonality and IgE production are curtailed.

### New therapeutic developments?

Different outlook, different aims—will these developments be translated into practical improvements which are lacking in traditional immunology? Someone has already said that: "Predictions are difficult, specially in relation to the future." We believe that global methods of analyses of Igs and, hopefully in the near future of T cells, will lead to the detection of distortions in the dynamics of the immune system which may be of clinical significance. The general picture created by the systemic outlook suggest that exploring the plasticity of lymphocytes networks aiming the restoration of a lost connectivity will bring more benefits that immunosuppression. Possibly, the use of large doses of intravenous IgG functions by restoring a lost connectivity. Initiatives of purifying anti-idiotypic Igs contained in IVIg in therapy of specific clinical conditions have been made and seem to be promising (Shoenfeld et al. 2002). T cell vaccination is another largely unexplored frontier, which has already been proved of some clinical utility (Krause et al. 1999; Li et al. 2005). A novel the treatment of severe bronchial asthma has been the use of a monoclonal anti-IgE antibody (Marcus 2006); its clinical efficacy warrants further investigation in its mechanism of action.

### Coda: From chance to history

As pointed out above, a random origin of variants is essential to maintain natural selection of living species as the guiding force in evolution and is also essential to maintain clonal selection of lymphocytes as the explanation of immunological activity. Random processes are polarly opposite to conservative, systemic/historical processes.

Our purpose in this essay has been to contrast the standard description of immunological activity with a way of seeing based on the biology of cognition and language, which is a general theory explaining the biological basis of human understanding and the nature of living systems proposed by Maturana et al. (Maturana 2002; Maturana and Poerksen 2004; Maturana and Varela 1980, 1987; Maturana and Mpodozis 1987, 2000). This entails a switch from a way of seeing based on randomness plus selection (Darwinism), to a description based on systemic/historical processes; a change from chance to history, where history is understood as a sequence of structural changes distinguished by a human observer. In this second way of seeing, conservation rather than variation plus selection, becomes the guiding notion. Evolution is no longer understood as a random process; rather phylogeny is seen as a natural *structural* drift.

Changes in a natural structural drifts are not random, but rather structurally-determined. A boat adrift, although it follows no route and is supposed to go anywhere, actually follows a single path perfectly determined by its size and weight, the force of the wind, waves, currents and other factors. Eventually, a boat adrift may collide with rocks and sink or reach a beach and stop drifting, but these particular events in time never play the role of references for the drifting. Thus, we cannot legitimately claim that, in its drifting, the boat "approached" the rocks or the beach where it stops drifting, because an event in the future cannot serve as reference for structural changes in the present (Maturana and Mpodozis 2000).

### References

Appleton AL, Curtis, Wilkes J, Cant AJ. 1994. Differentiation of materno-fetal GVHD from Omenn's syndrome in pre-BMT patients with severe combined immunodeficiency. Bone Marrow Transplant 14(1):157–159.

Bateson G. 1973. Steps to an ecology of mind. New York, NY: Ballantine Books.

Besredka A. 1909. De l'anaphylaxie. Sixiéme memoire de l'anaphylaxie lactique. Ann Inst Pasteur 23:166–174.

Billingham RE, Brent L, Medawar PB. 1953. Actively acquired tolerance of foreign cells. Nature 172:603–606.

Bos NA, Benner R, Wostmann BS, Pleasants JR. 1986. 'Background' Ig-secreting cells in pregnant germfree mice fed a chemically defined ultrafiltered diet. J Reprod Immunol 9(3):237–246.

Brandtzaeg P. 1996. History of oral tolerance and mucosal immunology. Ann NY Acad Sci 778:1–26.

Burnet FM. 1957. A modification of Jerne's theory of antibody production using the concept of clonal selection. Aust J Sci 20:67–69.

Caligiuri G, Stahl D, Kaveri S, Irinopoulous T, Savoie F, Mandet C, Vandaele M, Kazatchkine MD, Michel J-B, Nicoletti A. 2003. Autoreactive antibody repertoire is perturbed in atherosclerotic patients. Lab Invest 83(7):939–945.

Carvalho CR, Verdolin BA, Vaz NM. 1996. Indirect effects of oral tolerance cannot be ascribed to bystander suppression. Scand J Immunol 45:276–281.

Chase M. 1945. Cellular transfer of cutaneous hypersensitivity to tuberculin. Proc Soc Exp Biol Med 59:134–135.

Chase M. 1946. Inhibition of experimental drug allergy by prior feeding of sensitizing agents. Proc Soc Exp Biol Med 61:257–262.

Cohen IR, Sercarz W. 2004. Introduction: T cell degeneracy. Mol Immunol 40(14–15):983.

Corneo B, Moshous D, Gungor T, Wulffraat N, Philippet P, Le Deist FL, Fischer A, de Villartay JP. 2001. Identical mutations in RAG1 or RAG2 genes leading to defective V(D)J recombinase activity can cause either T–B-severe combined immune deficiency or Omenn syndrome. Blood 97(9):2772–2776.
Coutinho A, Kazatchkine MD, Avrameas S. 1995. Natural autoantibodies. Curr Opin Immunol 7(6):812–818.
Coutinho A. 2005. The Le Douarin phenomenon: A shift in the paradigm of developmental self-tolerance. Int J Dev Biol 49(2–3):131–136.
Davies JM, O'Hehir RE. 2004. VH gene usage in immunoglobulin E responses of seasonal rhinitis patients allergic to grass pollen is oligoclonal and antigen driven. Clin Exp Allergy 34:429–436.
de Lafaille MA, Muriglan S, Sunshine MJ, Lei Y, Kutchukhidze N, Furtado GC, Wensky AK, Olivares-Villagomez D, Lafaille JJ. 2001. Hyper immunoglobulin E response in mice with monoclonal populations of B and T lymphocytes. J Exp Med 194(9):1349–1359.
Duchman R, Schimit E, Knolle P, Meyer Zum K, Buschenfelde H, Markus Neurath M. 1996. Tolerance towards resident intestinal flora in mice is abrogated in experimental colitis and restored by treatment with interleukin-10 or antibodies to interleukin-12. Eur J Immunol 26:934–938.
Ehrlich P. 1900. On immunity, with special reference to cell life. Proc R Soc (Lond) 66(432):424–448.
Eisen HN. 2001. Specificity and degeneracy in antigen recognition: Yin and yang in the immune system. Annu Rev Immunol 19:1–21.
Faria AMC, Weiner HL. 2005. Oral tolerance. Immunol Rev 206: 232–259.
Ferreira C, Mouthon L, Nobrega A, Haury M, Kazatchkine MD, Ferreira E, Padua F, Coutinho A, Sundblad A. 1997. Instability of natural antibody repertoires in systemic lupus erythematosus patients, revealed by multiparametric analysis of serum antibody reactivities. Scand J Immunol 45(3):331–341.
Fesel C, Coutinho A. 1998. Dynamics of serum IgM autoreactive repertoires following immunization: Strain specificity, inheritance and association with autoimmune disease susceptibility. Eur J Immunol 28(11):3616–3629.
Fesel C, Goulart LF, Silva Neto A, Coelho A, Fontes CJF, Braga EM, Vaz NM. 2005. Increased polyclonal immunoglobulin reactivity toward human and bacterial proteins is associated with clinical protection in human plasmodium infection. Malar J 4:5–11.
Gowans JL. 1996. The lymphocyte—a disgraceful gap in medical knowledge. Immunol Today 17(6):288–291.
Guilherme L, Kalil J. 2004. Rheumatic fever: From sore throat to autoimmune heart lesions. Int Arch Allergy Immunol 134(1): 56–64.
Hashimoto K, Handa H, Umehara K, Sasaki S. 1978. Germfree mice reared on an "antigen-free" diet. Lab Anim Sci 28(1):38–45.
Haury M, Grandien A, Sundblad A, Coutinho A, Nobrega A. 1994. Global analysis of antibody repertoires. 1. An immunoblot method for the quantitative screening of a large number of reactivities. Scand J Immunol 39(1):79–87.
Haury M, Sundblad A, Grandien A, Barreau C, Coutinho A, Nobrega A. 1997. The repertoire of serum IgM in normal mice is largely independent of external antigenic contact. Eur J Immunol 27(6):1557–1563.
Jendro MC, Ganten T, Matteson EL, Weyand CM, Goronzy JJ. 1995. Emergence of oligoclonal T cell populations following therapeutic T cell depletion in rheumatoid arthritis. Arthritis Rheum 38(9):1242–1251.
Jerne NK. 1955. The natural selection theory of antibody formation. Proc Natl Acad Sci USA 41:849–857.
Jerne NK. 1974. Towards a network theory of the immune system. Ann Immunol 125C:373–392.
King C, Ilic A, Koelsch K, Sarvetnick N. 2004. Homeostatic expansion of T cells during immune insufficiency generates autoimmunity. Cell 117:265–277.
Krause I, Tomer Y, Elias D, Blank M, Gilburd B, Cohen IR, Shoenfeld Y. 1999. Inhibition of diabetes in NOD mice by idiotypic induction of SLE. J Autoimmun 13:49–55.
Lacroix-Desmazes S, Mouthon L, Kaveri SV, Kazatchkine MD. 1999. Stability of natural self-reactive antibody repertoires during aging. J Clin Immunol 19(1):26–34.
Lafaille JJ, Nagashima K, Katsuki M, Tonegawa S. 1994. High incidence of spontaneous autoimmune encephalomyelitis in immunodeficient anti-myelin basic protein T cell receptor transgenic mice. Cell 78(3):399–408.
Landsteiner K. 1901. The agglutinative properties of normal human blood. Wien Klin Wochenschr 14:424–448.
Launois P, Maillard I, Pingel S, Swihart KG, Xenarios I, Acha-Orbea H, Diggelmann H, Locksley RM, MacDonald HR, Louis JA. 1997. IL-4 rapidly produced by Vb4 Va8 CD41 T cells instructs Th2 development and susceptibility to leishmania major in BALBc mice. Immunity 6:541–549.
Levine BB, Vaz NM. 1970. Effect of combinations of inbred strains, antigen and antigen dose on immune responsiveness and reagin production in the mouse. Int Arch Allergy 39:156–164.
Li ZG, Mu R, Dai ZP, Gao XM. 2005. T cell vaccination in systemic lupus erythematosus with autologous activated T cells. Lupus 14(11):884–889.
Marcos RAM, De la Hera A, Gaspar ML, Marquez C, Bellas C, Mampaso F, Toribio ML, Martinez C-A. 1986. Modification of emerging repertoires by immunosuppression in immunodeficient mice results in autoimmunity. Immunol Rev 94:51–74.
Marcus P. 2006. Incorporating anti-IgE (omalizumab) therapy into pulmonary medicine practice: Practice management implications. Chest 129(2):466–474.
Margolis DA, Casper JT, Segura AD, Janczak T, McOlash L, Fisher B, Miller K, Gorski J. 2000. Infiltrating T cells during liver graft-versus-host disease show a restricted T-cell repertoire. Biol Blood Marrow Transplant 6(4):408–415.
Maturana HR. 1987. Everything is said by an observer. In: Thompson WI, editor. Gaia: A way of knowing. Political implications of the new biology. New York, NY: Lindisfarne Press.
Maturana H. 2002. Autopoiesis, structural coupling and cognition: A history of these and other notions in the biology of cognition. Cybern Hum Knowing (pdf available at www.matriztica.org). 9(3–4):5–34.
Maturana H. 2005. The origin and conservation of self-consciousness. Reflections on four questions by Heinz von Foerster. Kybernetes 34(1/2):54–88.
Maturana HR, Mpodozis J. 1987. Perception: Behavioral configuration of the object. Arch Biol Med Exp (Santiago) 20(3–4): 319–324.
Maturana H, Poerksen B. 2004. From being to doing: The origins of biology of cognition. Heidelberg: Carl-Auer.
Maturana HR, Varela FJ. 1980. Autopoiesis and cognition: The realization of living. Amsterdam: Reidel.
Maturana HR, Varela FJ. 1987. The tree of knowledge. Biological basis of human understanding. Boston: New Science Library.
Maturana HR. 1988. Reality: The search for objectivity or the quest for a compelling argument. Ir J Psychol 9(1):25–82.
Maturana HR. Comment on fedanzo Jr., Anthony 1983. All things are full of gods—or information. J Soc Biol Struct 6:155–158.
Maturana H, Mpodozis J. 2000. The origin of species by means of natural drift. Revista Chilena Hist Nat 73:261–310.
Maturana H. 2006. Autopoiésis y sistemas dinámicos cerrados: Conservación dinámica de la identidad sistémica, www.matriztica.org.
Mazumdar P. 1996. Species and specificity. An interpretation of the history of immunology. New York, NY: Cambrige University Press.
Menezes JS, Mucida DS, Cara DC, Alvarez-Leite JI, Russo M, Vaz NM, Faria AMC. 2003. Stimulation by food proteins plays critical role in the maturation of the immune system. Int Immunol 15(3):447–455.

Min B, McHugh R, Sempowski GD, Mackall C, Foucras F, Paul WE. 2003. Neonates support lymphopenia-induced lymphopoiesis. Immunity 18:131–140.

Mouthon L, Nobrega A, Nicolas N, Kaveri SV, Barreau C, Coutinho A, Kazatchkine MD. 1995. Invariance and restriction toward a limited set of self-antigens characterize neonatal IgM antibody repertoires and prevail in autoreactive repertoire of healthy adults. Proc Natl Acad Sci USA 92:3839–3843.

Murata H, Matsumura R, Koyama A, Sugiyama T, Sueishi M, Shibuya K, Tsutsumi A, Sumida T. 2002. T cell receptor repertoire of T cells in the kidneys of patients with lupus nephritis. Arthritis Rheum 46(8):2141–2147.

Nóbrega A, Haury M, Grandien A, Malanchere E, Sundblad A, Coutinho A. 1993. Global analysis of antibody repertoires. II. Evidence for specificity, self-selection and the immunological "homunculus" of antibodies in normal serum. Eur J Immunol 23(11):2851–2859.

Olivares-Villagomez D, Wensky AK, Wang Y, Lafaille JJ. 2000. Repertoire requirements of CD4+ T cells that prevent spontaneous autoimmune encephalomyelitis. J Immunol 164(10):5499–5507.

Orsini E, Alyea EP, Schlossman R, Canning C, Soiffer RJ, Chillemi A, Neuberg D, Anderson KC, Ritz J. 2000. Changes in T cell receptor repertoire associated with graft-versus-tumor effect and graft-versus-host disease in patients with relapsed multiple myeloma after donor lymphocyte infusion. Bone Marrow Transplant 25(6):623–632.

Pasteur L. 1878. The germ theory and its application to medicine and surgery. Comp Rend l'acad Sci 86:1037–1043.

Pereira P, Larsson E-L, Forni L, Bandeira A, Coutinho A. 1985. Natural effector T lymphocytes in normal mice. Proc Natl Acad Sci USA 82:7691–7695.

Pingel S, Launois P, Fowell DJ, Turck CW, Southwood S, Sette A, Glaichenhaus N, Louis JA, Locksley RM. 1999. Altered ligands reveal limited plasticity in the T cell response to a pathogenic epitope. J Exp Med 189(7):1111–1120.

Quintana FJ, Hagedorn PH, Elizur G, Merbl Y, Domany E, Cohen IR. 2004. Functional immunomics: Microarray analysis of IgG autoantibody repertoires predicts the future response of mice to induced diabetes. Proc Natl Acad Sci USA 101(Suppl 2):14615–14621.

Sakaguchi S. 2005. Naturally arisinf Foxp3-expressing CD25+ CD4+ regulatory T cells in immunologicaltoleance to self and non-self. Nat Immunol 6(4):345–352.

Sakkas LI, Xu B, Artlett CM, Lu S, Jimenez SA, Platsoucas CD. 2002. Oligoclonal T cell expansion in the skin of patients with systemic sclerosis. J Immunol 168(7):3649–3659.

Sekine T, Kato R, Kato T, Masuko-Hongo K, Kameko F, Maruyama M, Nishioka K, Yamamoto K. 2000. Accumulation of identical T cell clones in the right and left lobes of the thyroid gland in patients with Graves' disease: Analysis of T cell clonotype *in vivo*. Endocr J 47(2):127–136.

Shoenfeld Y, Rauova L, Gilburd B, Kvapil F, Goldberg I, Kopolovic J, Rovensky J, Blank M. 2002. Efficacy of IVIG affinity-purifed anti-doublestranded DNA anti-idiotypic antibodies in the treatment of an experimental murine model of systemic lupus erythematosus. Int Immunol 14(11):1303–1311.

Simonsen M. 1962. Graft versus host reactions. Their natural history, and applicability as tools of research. Prog Allergy 6:349–467.

Stahl D, Lacroix-Desmazes S, Mouthon L, Kaveri SV, Kazatchkine MD. 2000. Analysis of human self-reactive antibody repertoires by quantitative immunoblotting. J Immunol Methods 240(1–2): 1–14.

Stockinger B, Kassiotis G, Bourgeois C. 2004. Homeostasis and T cell regulation. Curr Opin Immunol 16:775–779.

Talmage DW. 1959. Immunological specificity, unique combinations of selected natural globulins provide an alternative to the classical concept. Science 129:1643–1648.

Tauber AI. 1997. Historical and philosophical perspectives concerning immune cognition. J Hist Biol 30(3):419–440.

Troy AE, Shen H. 2003. Cutting edge: Homeostatic proliferation of peripheral T lymphocytes is regulated by clonal competition. J Immunol 170(2):672–676.

Vakil M, Sauter J, Paige C, Kearney JF. 1986. *In vivo* suppression of perinatal multispecific B cells results in a distortion of the adult B cell repertoire. Eur J Immunol 16:1159–1165.

Vasconcellos R, Nobrega A, Haury M, Viale AC, Coutinho A. 1998. Genetic control of natural antibody repertoires: I. IgH, MHC and TCR beta loci. Eur J Immunol 28(3):1104–1115.

Vaz NM, Carvalho CR. 1993. Immunological specificity as metaphor. Braz J Med Biol Res 26:665–671.

Vaz N, Ramos GC. 2006. Immunity and intentionality: The specificity of immunological observations, submitted.

Vaz NM, Varela FJ. 1978. Self and nonsense; an organism-centered approach to immunology. Med Hypotheses 4:238–253.

Vaz NM, Faria AMC, Verdolin BA, Carvalho CR. 1997. Immaturity, ageing and oral tolerance. Scand J Immunol 46:225–229.

Vaz N, Silva Neto A, Verdolin B, Coelho A, Correa-Oliveira R, Braga E, Fesel C. 2000. Perfis de IgM e IgG naturais em parasitoses crônicas humanas graves XV Reunião da fesbe., p 166, (129.007). Caxambu, MG.

Vaz NM, Fesel C, Nobrega A, Silva Neto AF, Secor WE, Colley DG. 2001. Severity of schistosomiasis mansoni in male CBA mice is related to IgG profiles reacting with mouse liver extracts in Panama-blots XVI Reunião annual da fedbe., p 136, (124.003). Caxambu MG.

Vaz N, Faria AMC, Menezes JS, Verdolin BA, Silva Neto AF, Carvalho CR. 2003. The conservative physiology of the immune system. Braz J Med Biol Res 36:13–22.

Verdolin BA, Ficker SM, Faria AM, Vaz NM, Carvalho CR. 2001. Stabilization of serum antibody responses triggered by initial mucosal contact with the antigen independently of oral tolerance induction. Braz J Biol Med Res 34(2):211–219.

Villa A, Santagata S, Bozzi F, Imberti L, Notarangelo LD. 1999. Omenn syndrome: A disorder of Rag1 and Rag2 genes. J Clin Immunol 19(2):87–97.

Wang L, Tadokoro K, Tokunaga K, Uchida S, Moriyama S, Bannai M, Mitsunaga S, Takai K, Juji T. 1997. Restricted use of T-cell receptor V beta genes in posttransfusion graft-versus-host disease. Transfusion 37(11-12):1184–1191.

Wells HG. 1911. Studies on the chemistry of anaphylaxis. III. Experiments with isolated proteins, specially those of the hen's egg. J Infect Dis 9:147–171.

Wu Z, Bensinger SJ, Zhang J, Chen C, Yuan X, Huang X, Markmann JF, Kassaee A, Rosengard BR, Hancock WW, Sayegh MH, Turka LA. 2004. Homeostatic proliferation is a barrier to transplantation tolerance. Nat Med 10(1):87–92.

